# Additional cytogenetic abnormalities in patients with newly diagnosed acute promyelocytic leukemia predict inferior event‐free survival

**DOI:** 10.1002/cam4.6398

**Published:** 2023-08-16

**Authors:** Hui Zeng, Hai‐Bo Dong, Qi‐Guo Zhang, Min Zhou, Qian Zhang, Lan‐Xin Chen, Cui‐Ying Yuan, Ru‐Ru Jiang, Jin‐Wen Liu, Jian Ou‐Yang, Jie He, Bing Chen

**Affiliations:** ^1^ Department of Hematology Nanjing Drum Tower Hospital, Affiliated Hospital of Medical School, Nanjing University Nanjing China

**Keywords:** acute promyelocytic leukemia, additional cytogenetic abnormalities, event‐free survival, prognosis

## Abstract

**Background:**

The innovative combination of all‐trans retinoic acid (ATRA) and arsenic trioxide (ATO) has established a new chapter of curative approach in acute promyelocytic leukemia (APL). The disease characteristics and prognostic influence of additional cytogenetic abnormalities (ACA) in APL with modern therapeutic strategy need to be elucidated.

**Methods:**

In the present study, we retrospectively investigated disease features and prognostic power of ACA in 171 APL patients treated with ATRA‐ATO‐containing regimens.

**Results:**

Patients with ACA had markedly decreased hemoglobin levels than that without ACA (*p* = 0.021). Risk stratification in the ACA group was significantly worse than that in the non‐ACA group (*p* = 0.032). With a median follow‐up period of 62.0 months, worse event‐free survival (EFS) was demonstrated in patients harboring ACA. Multivariate analysis showed that ACA was an independent adverse factor for EFS (*p* = 0.033). By further subgroup analysis, in CD34 and CD56 negative APL, patients harboring ACA had inferior EFS (*p* = 0.017; *p* = 0.037).

**Conclusions:**

To sum up, ACA remains the independent prognostic value for EFS, we should build risk‐adapted therapeutic strategies in the long‐term management of APL when such abnormalities are detected.

## 1 INTRODUCTION

Acute promyelocytic leukemia (APL) is a special disease entity of acute myeloid leukemia.^1^ The hallmark of this disease is characterized by specific balanced reciprocal translocation, namely *t* (15;17), however, approximately 26%–39% of patients exhibit one or more additional cytogenetic abnormalities (ACA) beyond *t* (15;17).^2^ To date, the prognostic relevance of ACA in APL still has remained a matter of debate.^3^ Some studies^4^, ^5^, ^6^, ^7^ have shown no correlations were found between ACA and clinical parameters such as Sanz's risk category, initial white blood cell (WBC) counts, and microgranular morphology (M3v). Clinical prognosis was also similar between patients with or without ACA for overall survival (OS) and progression‐free survival (PFS).^8^, ^9^ However, other contradictory researches have observed the presence of ACA strongly associated with bcr3 isoform, higher incidence of differentiation syndrome (DS), and greater risk of induction death. ACA had the independent power to predict inferior OS and PFS.^7^, ^10^ Recently, all‐trans retinoic acid (ATRA)‐arsenic trioxide (ATO) combination first‐line therapy has formed the current standard of care, however, most previous studies exploring ACA and clinical outcomes either included patients treated without ATO or analyzed ATO‐ and non‐ATO‐treated patients together.^11^ In order to clarify the role of ACA in contemporary therapeutic strategy, here we investigated the clinicopathological characteristics and prognostic effect of cytogenetics in a series of 171 successfully karyotyped patients with a long follow‐up.

## 2 PATIENTS AND METHODS

### 2.1 Patients

Between January 2010 and May 2022, adult patients with newly diagnosed APL were registered with this study. Patients were assessed for eligibility for enrollment based on the following criteria: Diagnosis of APL with *t* (15;17) and or the *PML::RARα* fusion gene amplified by reverse transcriptase polymerase chain reaction (RT‐PCR); With a complete set of clinical information and follow‐up data including chromosome karyotype.

### 2.2 Methods

#### 2.2.1 Cytogenetics analysis

Bone marrow samples for cytogenetic analysis were processed after 24‐h culture following standard procedures. The chromosomes were stained by R‐banding and the karyotypes were designated according to International System for Human Cytogenetic Nomenclature (ISCN, 1995) recommendations.^12^ Twenty or more metaphases were fully analyzed. Cases were considered normal diploid if no clonal abnormalities were detected in at least 20 mitotic cells. In patients with normal karyotype, additional testing was carried out to detect *PML::RARα* rearrangement by RT‐PCR and fluorescence in situ hybridization (FISH) on metaphase and interphase nuclei. Dual‐color FISH was performed using a *PML::RARα* translocation probe.

#### 2.2.2 Variables collection

Basic pretreatment variables, consisting of sex, age, Eastern Cooperative Oncology Group (ECOG) performance status score, complete blood counts, lactate dehydrogenase (LDH) level, albumin, triglyceride, cholesterol, activated partial thromboplastin time (APTT), prothrombin time (PT), fibrinogen, D‐dimer, promyelocyte percentage in bone marrow (BM) and peripheral blood (PB), immunophenotype (CD2/CD5/CD7/CD11b/CD15/CD19/CD34/CD56/CD64/CD117/.

HLA‐DR positive ≥ 20%), Sanz's score category, *FLT3* mutation, *PML::RARα* isoform (definition by RT‐PCR), positive rate of *PML::RARα* fusion (identification by FISH), FAB subtype, body mass index (BMI), disseminated intravascular coagulation (DIC) score, DS, molecular complete remission (mCR), QT prolongation, second primary malignancy (SPM), internal bleeding, and embolism were analyzed.

### 2.3 Chromosome karyotype subgroup

ACA subgroup was defined as one or more additional cytogenetic abnormalities besides only *t* (15;17). The non‐ACA subgroup included normal karyotype, *t* (15;17) as the sole chromosomal abnormality and other clonal abnormality.

### 2.4 Treatment

All patients received treatment according to guidelines set by the Hematological Society of the Chinese Medical Association. From 2010 to 2018, 70 patients were, respectively, given ATRA (20 mg/m^2^/day, until CR) and anthracycline‐based chemotherapy, ATRA and ATO (0.16 mg/kg/day, until CR) regimen, ATRA, ATO, and anthracycline‐based treatment. Remission induction was followed by two‐three chemotherapy consolidation courses with anthracycline ± Ara‐C regimens. Maintenance treatment (each cycle comprised ATAR and ATO) continued for 2 years and consisted of at least five cycles of 3 months each (for low‐intermediate risk patients). For high‐risk patients, each cycle consisted of intermittent ATRA, ATO, and continuous oral methotrexate (15 mg/m^2^ qw for 4 weeks), combined or not with 6‐mercaptopurine (50 mg/m^2^/day for 2–4 weeks). From 2018 onward, 101 patients experienced induction therapy with ATRA (25 mg/m^2^/day, until CR) and ATO (0.16 mg/kg/day, until CR). Hydroxycarbamide was administered when WBC count rose >4 × 10^9^/L; idarubicin (8 mg/m^2^/day on days 1–3) or daunorubicin (45 mg/m^2^/day on days 1–3) was given when WBC count increased >10 × 10^9^/L. Post‐remission therapy was followed by seven/four consolidation cycles with ATRA and ATO regimen (for low‐intermediate risk patients). For high‐risk patients, remission induction was followed by three consolidation cycles with anthracycline plus Ara‐C regimens, maintenance treatment (each cycle comprised ATAR and ATO) lasted 2 years and comprised at least eight cycles of 3 months each. 10 mg dexamethasone twice daily intravenously was given to patients with DS. Blood and fibrinogen product support were applicated to maintain platelet count above 40 × 10^9^/L, and fibrinogen concentration above 1.5 g/L during induction.

### 2.5 Study definitions and endpoints

Early death was defined as death within the first 30 days of presentation to medical care. Relapse and mCR were defined by NCI criteria.^13^ SPM was defined as a metachronous malignancy developing at least 6 months after the diagnosis of APL.^14^ QT prolongation corrected for heart rate was defined as longer than 450 ms (male) or 470 ms (female). DIC scores were calculated according to the definition of the International Society on Thrombosis and Hemostasis.^15^ Internal bleeding was defined as bleeding other than mucocutaneous hemorrhage. Thrombotic events were confirmed by computer tomography scan, Doppler ultrasound, and magnetic resonance imaging examination. OS was defined as the time from diagnosis to death or last follow‐up. PFS was defined as the time from diagnosis to disease relapse, death or last follow‐up (Bone marrow relapse was defined as the reappearance of RT‐PCR positivity in two consecutive samples taken at one‐month intervals at any time. Central nervous system relapse was confirmed by lumbar puncture and cytologic examination of cerebrospinal fluid). Event‐free survival (EFS) was defined as the time from mCR to any event including SPM, QT prolongation, internal bleeding, disease recurrence, and death.

### 2.6 Statistical methods

Statistical analysis was performed by GraphPad Prism 8 and SPSS 23.0 software. Continuous variables were expressed as mean ± standard deviation or median (range), according to whether the data conform to normal distribution. Continuous variables were analyzed by *t*‐test or Mann⁃Whitney *U*‐test, and categorical variables were analyzed by Fisher exact test. The Kaplan–Meier survival curve was used to analyze the survival outcomes. The prognostic factors of OS, PFS, and EFS were analyzed by univariate and multivariate Cox regression. *p* < 0.050 was considered statistically significant.

## 3 RESULTS

### 3.1 Baseline clinical characteristics

**FIGURE 1:**
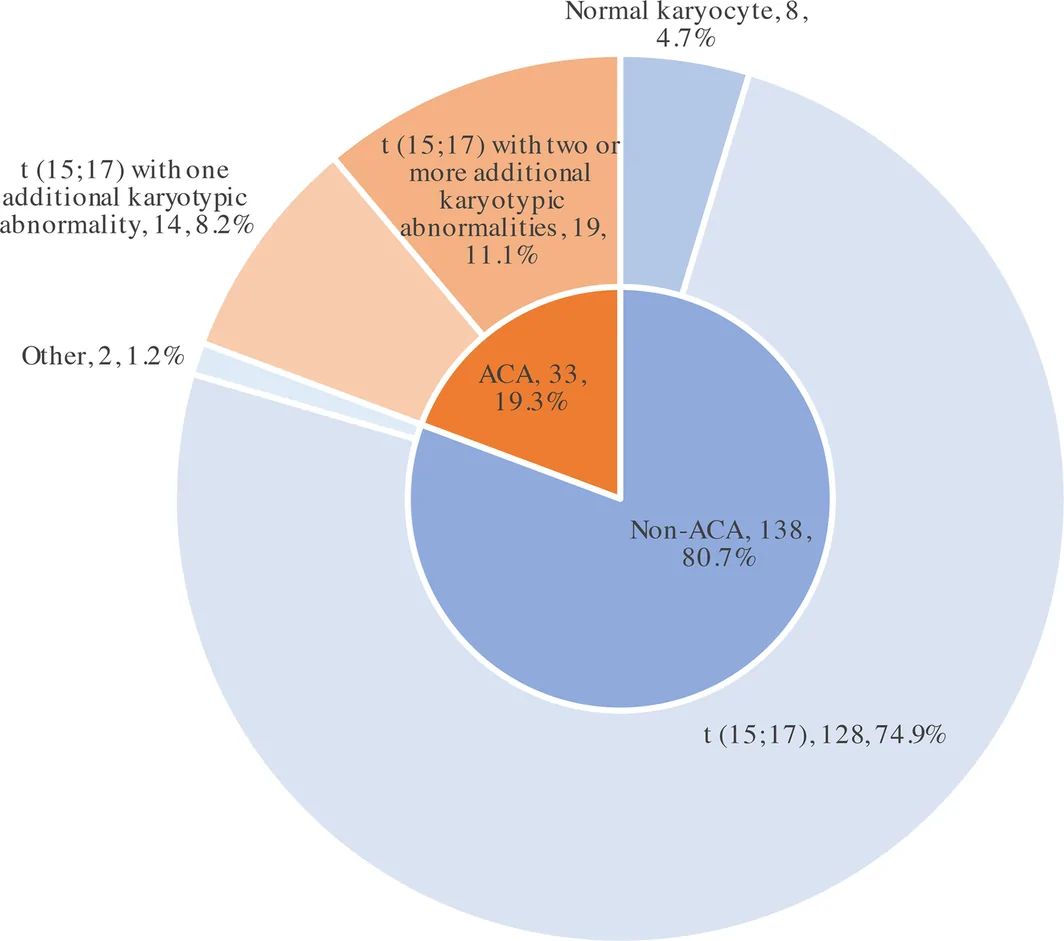
Karyotype distribution of 171 APL patients.

A total of 171 patients with APL were enrolled in this study. The median age was 42.0 (range 16.0–75.0) years, including 75 males (43.9%) and 96 females (56.1%). ACA was observed in 33 patients (19.3%), 14 of the cases had one additional abnormality (8.2%) and 19 had two or more abnormalities (11.1%). In total, 138 patients (80.7%) involved non‐ACA changes, of which, an isolated *t* (15;17) karyotype was seen in 128 cases (74.9%), normal karyotype was detected in 8 cases (4.7%), 1 case (0.6%) had normal karyotype accompanying trisomy 8 and 1 case (0.6%) had normal karyotype accompanying chromosome 21 (Figure 1).

Patients with ACA had markedly decreased hemoglobin levels than patients without ACA (*p* = 0.021), and the risk stratification in the ACA group was significantly worse than that in the non‐ACA group (*p* = 0.032). Compared with the non‐ACA subgroup, there were no significant differences in sex, age, ECOG performance status score, complete blood counts, LDH, albumin, triglyceride, cholesterol, APTT, PT, fibrinogen, D‐dimer, BMI, BM and PB promyelocyte percentage, immunophenotype, *FLT3* mutation, *PML::RARα* isoform subtype, positive rate of *PML::RARα* fusion, FAB type, and DIC scores (all *p* > 0.050) (Table 1). Moreover, no significant differences were observed within the subgroup (all *p* > 0.050).

**TABLE 1:** Baseline characteristics of patients with non‐ACA and ACA.

	Non‐ACA group, *N* = 138	ACA group, *N* = 33	*p*‐value
Sex, male [*n* (%)]	60 (43.5)	15 (45.5)	0.848
Age (years), *M* (range)	42.5 (16.0, 75.0)	40.0 (18.0, 65.0)	0.769
ECOG PS ≥ 2, [*n* (%)]	59 (42.8)	18 (54.5)	0.247
White blood cell count (10^9^/L), *M* (range)	2.15 (0.20, 125.80)	4.30 (0.50, 140.20)	0.098
Hemoglobin (g/L), *M* (range)	96.0 (45.0, 157.0)	82.0 (44.0, 129.0)	**0.021**
Platelets (10^9^/L), *M* (range)	28.0 (4.0, 157.0)	31.0 (4.0, 155.0)	0.823
LDH (U/L), *M* (range)	278.5 (115.0, 1281.0)	319.0 (131.0, 4679.0)	0.217
Albumin (g/L), *M* (range)	40.8 (29.0, 50.2)	40.3 (27.7, 48.3)	0.600
Triglyceride (mmol/L), *M* (range)	1.66 (0.53, 7.13)	2.00 (0.78, 5.83)	0.452
Cholesterol (mmol/L), *M* (range)	4.26 (1.98, 8.87)	4.03 (1.18, 6.29)	0.505
APTT (s), *M* (range)	26.8 (17.0, 59.2)	27.1 (20.8, 39.9)	0.918
PT (s), *M* (range)	13.5 (9.8, 23.0)	13.9 (11.6, 23.1)	0.354
Fibrinogen (g/L), *M* (range)	1.20 (0.20, 5.40)	1.20 (0.40, 4.30)	0.814
D‐dimer (mg/L), *M* (range)	6.54 (0.10, 82.25)	8.21 (0.13, 77.23)	0.197
Promyelocytic ratio (bone marrow), *M* (range)	84.0 (20.5, 99.0)	84.5 (48.0, 97.0)	0.728
Promyelocytic ratio (peripheral blood), *M* (range)	60.0 (1.0, 98.0)	53.0 (4.0, 96.0)	0.984
CD2 positive, [*n* (%)]	13 (9.4)	2 (6.1)	0.739
CD7 positive, [*n* (%)]	8 (5.8)	2 (6.1)	1.000
CD11b positive, [*n* (%)]	18 (13.0)	1 (3.0)	0.128
CD15 positive, [*n* (%)]	19 (13.8)	3 (9.1)	0.575
CD19 positive, [*n* (%)]	3 (2.2)	2 (6.1)	0.247
CD34 positive, [*n* (%)]	18 (13.0)	8 (24.2)	0.174
CD56 positive, [*n* (%)]	13 (9.4)	4 (12.1)	0.745
CD64 positive, [*n* (%)]	66 (47.8)	13 (39.4)	0.440
CD117 positive, [*n* (%)]	129 (93.5)	28 (84.8)	0.149
HLA‐DR positive, [*n* (%)]	14 (10.1)	2 (6.1)	0.539
Risk group, [*n* (%)]			**0.032**
Low‐risk	43 (31.2)	3 (9.1)	
Intermediate‐risk	36 (26.1)	13 (39.4)	
High‐risk	59 (42.8)	17 (51.5)	
*FLT3* mutation, [*n* (%)]			1.000
Negative	107 (77.5)	25 (75.8)	
Positive	31 (22.5)	8 (24.2)	
*PML::RARα* isoform, [n (%)]			0.124
BCR1	76 (55.1)	13 (39.4)	
BCR2	17 (12.3)	3 (9.1)	
BCR3	45 (32.6)	17 (51.5)	
positive rate of *PML::RARα* fusion, *M* (range)	80.0 (1.0, 98.2)	79.0 (35.0, 98.0)	0.395
FAB type, [*n* (%)]			0.626
3a	102 (73.9)	26 (78.8)	
3b	31 (22.5)	7 (21.2)	
3v	5 (3.6)	0 (0.0)	
BMI (kg/m^2^), *M* (range)	23.14 (17.19, 34.37)	23.23 (16.60, 32.65)	0.910
DIC scores, [*n* (%)]			0.372
<5	18 (13.0)	2 (6.1)	
≥5	120 (87.0)	31 (93.9)	

*Note*: Significant p‐value is in boldface.

### 3.2 Early events

Based on the time to mCR, clinical events were divided into early events (DS, QT prolongation, internal bleeding, embolism, and early death) and late events (SPM, QT prolongation, bleeding, disease recurrence, and death). During induction remission, 41 patients had DS (34 with non‐ACA and 7 with ACA, *p* = 0.822). QT prolongation was observed in 5 patients (4 with non‐ACA and 1 with ACA, *p* = 1.000). In total, 25 patients occurred with internal bleeding (19 with non‐ACA and 6 with ACA, *p* = 0.583). In total, 7 patients had embolisms (5 with non‐ACA and 2 with ACA, *p* = 0.621). Early death was identified in 9 patients (6 with non‐ACA and 3 with ACA, *p* = 0.378). The incidence of early events and the time to mCR were not different between the two subgroups (*p* > 0.050, shown in Table 2). The remaining 162 patients received mCR after induction therapy, there were no primary resistant patients in the two subgroups.

**TABLE 2:** Comparison of early events between non‐ACA and ACA patients.

	Non‐ACA group, *N* = 138	ACA group, *N* = 33	*p‐*value
Differentiation syndrome, [*n* (%)]	34 (24.6)	7 (21.2)	0.822
QT prolongation, [*n* (%)]	4 (2.9)	1 (3.0)	1.000
Bleeding events, [*n* (%)]	19 (13.8)	6 (18.2)	0.583
Embolic events, [*n* (%)]	5 (3.6)	2 (6.1)	0.621
Death incident, [*n* (%)]	6 (4.3)	3 (9.1)	0.378
Time to mCR (days), M(range)	39.0 (28.0, 78.0)	38.0 (30.0, 67.0)	0.771

### 3.3 Late events

In total, 7 patients had SPM (4 with non‐ACA and 3 with ACA, *p* = 0.119), 2 patients (both ACA) with uterine teratoma, 2 patients (respectively, 1 non‐ACA and 1 ACA) with lung cancer, 1 patient (non‐ACA) with parathyroid adenoma, 1 patient (non‐ACA) with gastric cancer and 1 patient (non‐ACA) with nasopharyngeal carcinoma. QT prolongation was observed in 3 patients (2 with non‐ACA and 1 with ACA, *p* = 1.000). In total, 2 patients occurred gastrointestinal bleeding (respectively, 1 non‐ACA and 1 ACA, *p* = 0.337). Disease recurrence was detected in 7 patients (4 with non‐ACA and 3 with ACA, *p* = 0.119), primarily in the marrow, *n* = 6 (respectively, 3 non‐ACA and 3 ACA); 1 with central nervous system involvement (non‐ACA). In total, 5 patients received mCR after salvage therapy, and 2 patients (respectively, 1 non‐ACA and 1 ACA, both medullary relapse) died of disease progression. Late death occurred in 7 patients (5 with non‐ACA and 2 with ACA, *p* = 0.614) (Table 3). The incidence of late events was not different between the two subgroups.

**TABLE 3:** Comparison of late events between non‐ACA and ACA patients.

	Non‐ACA group, *N* = 132	ACA group, *N* = 30	*p‐*value
Secondary primary malignancy, [*n* (%)]	4 (3.0)	3 (10.0)	0.119
QT prolongation, [*n* (%)]	2 (1.5)	1 (3.3)	1.000
Bleeding events, [*n* (%)]	1 (0.8)	1 (3.3)	0.337
Disease recurrence events, [*n* (%)]	4 (3.0)	3 (10.0)	0.119
Death incident, [*n* (%)]	5 (3.8)	2 (6.7)	0.614

### 3.4 Prognostic analysis

**FIGURE 2:**
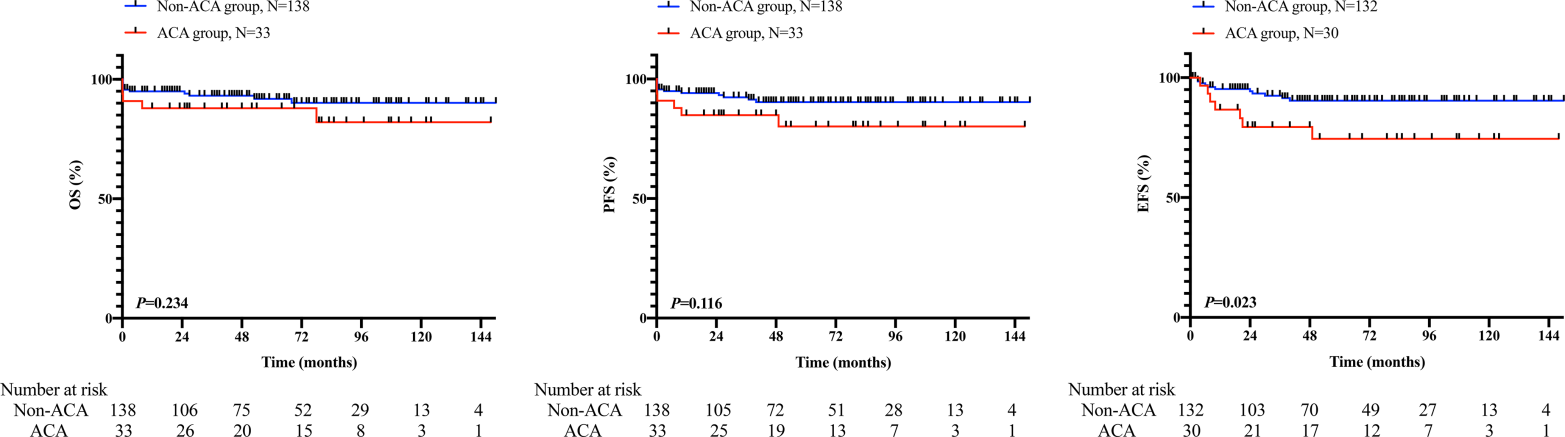
Kaplan–Meier survival analysis of 171 APL patients. EFS, event‐free survival; OS, overall survival; PFS, progression‐free survival.

With a median follow‐up of 62.0 months, the median OS, PFS, and EFS were not reached. In the non‐ACA subgroup, the cumulative disease recurrence rate at 1‐, 5‐, and 10‐year was 0.7%, 2.9%, and 2.9%, respectively. The 1‐, 5‐, and 10‐year cumulative incidence of disease recurrence in the ACA subgroup was 6.7%, 10.0%, and 10.0%, respectively (*p* > 0.050). Compared with the non‐ACA subgroup, the ACA subgroup had worse EFS (*p* = 0.023) (Figure 2). Univariate Cox regression analysis was used to explore the prognostic factors of OS, PFS, and EFS. The results showed that ACA was a poor prognostic factor for EFS (*p* = 0.030) (Table 4). The significant factors in univariate analysis were substituted into multivariate analysis, and the results showed that ACA was an independent prognostic factor for EFS (*p* = 0.033) (Table 5).

**TABLE 4:** Univariate Cox regression analysis.

	OS		PFS		EFS	
	*p‐*value	HR (95%CI)	*p‐*value	HR (95%CI)	*p‐*value	HR (95%CI)
Sex, female	**0.037**	0.325 (0.113–0.935)	**0.036**	0.349 (0.131–0.931)	0.847	1.098 (0.425–2.834)
Age (years)	0.850	0.997 (0.964–1.031)	0.774	0.995 (0.964–1.028)	0.723	1.006 (0.974–1.038)
ECOG PS ≥ 2	**0.021**	3.785 (1.220–11.740)	**0.019**	3.424 (1.220–9.606)	0.809	1.121 (0.442–2.841)
White blood cell count (10^9^/L)	0.404	1.007 (0.991–1.023)	0.424	1.006 (0.991–1.022)	0.645	1.004 (0.988–1.020)
Hemoglobin (g/L)	0.918	0.999 (0.980–1.018)	0.908	0.999 (0.981–1.017)	0.859	0.998 (0.980–1.017)
Platelets (10^9^/L)	0.212	0.986 (0.964–1.008)	0.142	0.984 (0.963–1.005)	0.565	0.995 (0.980–1.011)
Albumin (g/L)	0.656	1.028 (0.911–1.159)	0.285	1.066 (0.948–1.199)	0.438	1.046 (0.933–1.173)
LDH (U/L)	**<0.001**	1.001 (1.001–1.002)	**<0.001**	1.001 (1.001–1.002)	0.684	1.000 (0.999–1.002)
Triglyceride (mmol/L)	0.356	1.190 (0.822–1.723)	0.607	1.102 (0.760–1.599)	0.427	0.819 (0.501–1.340)
Cholesterol (mmol/L)	0.817	1.056 (0.667–1.671)	0.669	1.097 (0.718–1.677)	0.499	1.151 (0.766–1.731)
Fibrinogen (g/L)	0.095	0.510 (0.231–1.124)	0.09	0.530 (0.254–1.105)	0.545	0.840 (0.479–1.476)
D‐dimer (mg/L)	**0.002**	1.037 (1.013–1.062)	**0.001**	1.038 (1.014–1.062)	**0.003**	1.042 (1.014–1.070)
APTT (s)	**0.048**	1.062 (1.001–1.128)	**0.018**	1.068 (1.011–1.129)	0.140	1.049 (0.984–1.118)
PT (s)	**<0.001**	1.359 (1.164–1.587)	**<0.001**	1.347 (1.162–1.562)	0.146	1.154 (0.951–1.401)
CD2 positive	0.082	3.082 (0.867–10.961)	0.154	2.472 (0.713–8.573)	0.509	1.643 (0.376–7.181)
CD7 positive	0.139	3.098 (0.694–13.839)	0.235	2.441 (0.559–10.652)	0.825	1.256 (0.167–9.464)
CD11b positive	0.627	1.450 (0.324–6.487)	0.88	1.120 (0.257–4.888)	0.244	2.096 (0.603–7.284)
CD15 positive	0.362	0.390 (0.051–2.955)	0.293	0.339 (0.045–2.551)	0.249	0.305 (0.041–2.297)
CD19 positive	0.666	0.048 (0.000–47255.505)	0.646	0.048 (0.000–20747.076)	0.403	2.375 (0.313–18.022)
CD34 positive	**0.019**	3.363 (1.222–9.256)	**0.006**	3.758 (1.454–9.711)	**0.032**	2.925 (1.096–7.802)
CD56 positive	**0.032**	3.471 (1.113–10.825)	**0.008**	4.068 (1.440–11.493)	**<0.001**	6.679 (2.471–18.055)
CD64 positive	0.433	0.651 (0.223–1.901)	0.434	0.675 (0.252–1.808)	0.368	1.533 (0.604–3.888)
CD117 positive	**0.002**	0.187 (0.065–0.539)	**0.003**	0.205 (0.073–0.577)	0.448	0.566 (0.130–2.462)
HLA‐DR positive	0.513	1.644 (0.371–7.290)	0.713	1.319 (0.303–5.747)	0.400	0.043 (0.000–64.279)
Risk group, high‐risk	0.052	2.640 (0.990–7.036)	**0.029**	2.796 (1.109–7.050)	0.425	1.490 (0.559–3.973)
*FLT3* mutation	**0.021**	3.232 (1.198–8.718)	0.051	2.577 (0.997–6.662)	0.868	0.900 (0.260–3.111)
*PML::RARα* isoform, type 3	**0.032**	3.024 (1.099–8.324)	**0.024**	2.969 (1.150–7.665)	0.354	1.552 (0.612–3.934)
Chromosomal karyotype, ACA	0.243	1.879 (0.652–5.415)	0.126	2.151 (0.807–5.731)	**0.030**	2.861 (1.109–7.385)
FAB type, 3v	0.325	2.771 (0.365–21.040)	0.424	2.278 (0.303–17.145)	0.646	0.048 (0.000–20806.047)
BMI (kg/m^2^)	0.083	1.126 (0.985–1.288)	0.080	1.119 (0.987–1.268)	0.611	1.036 (0.904–1.186)

*Note*: Significant p‐value is in boldface.

**TABLE 5:** Multivariate Cox regression analysis.

	EFS	
	*p‐*value	HR (95%CI)
D‐dimer (mg/L)	**0.044**	1.033 (1.001–1.065)
CD34 positive	0.156	2.070 (0.757–5.661)
CD56 positive	**0.001**	5.705 (2.032–16.020)
Chromosomal karyotype, ACA	**0.033**	2.882 (1.091–7.609)

*Note*: Significant p‐value is in boldface.

**FIGURE 3:**
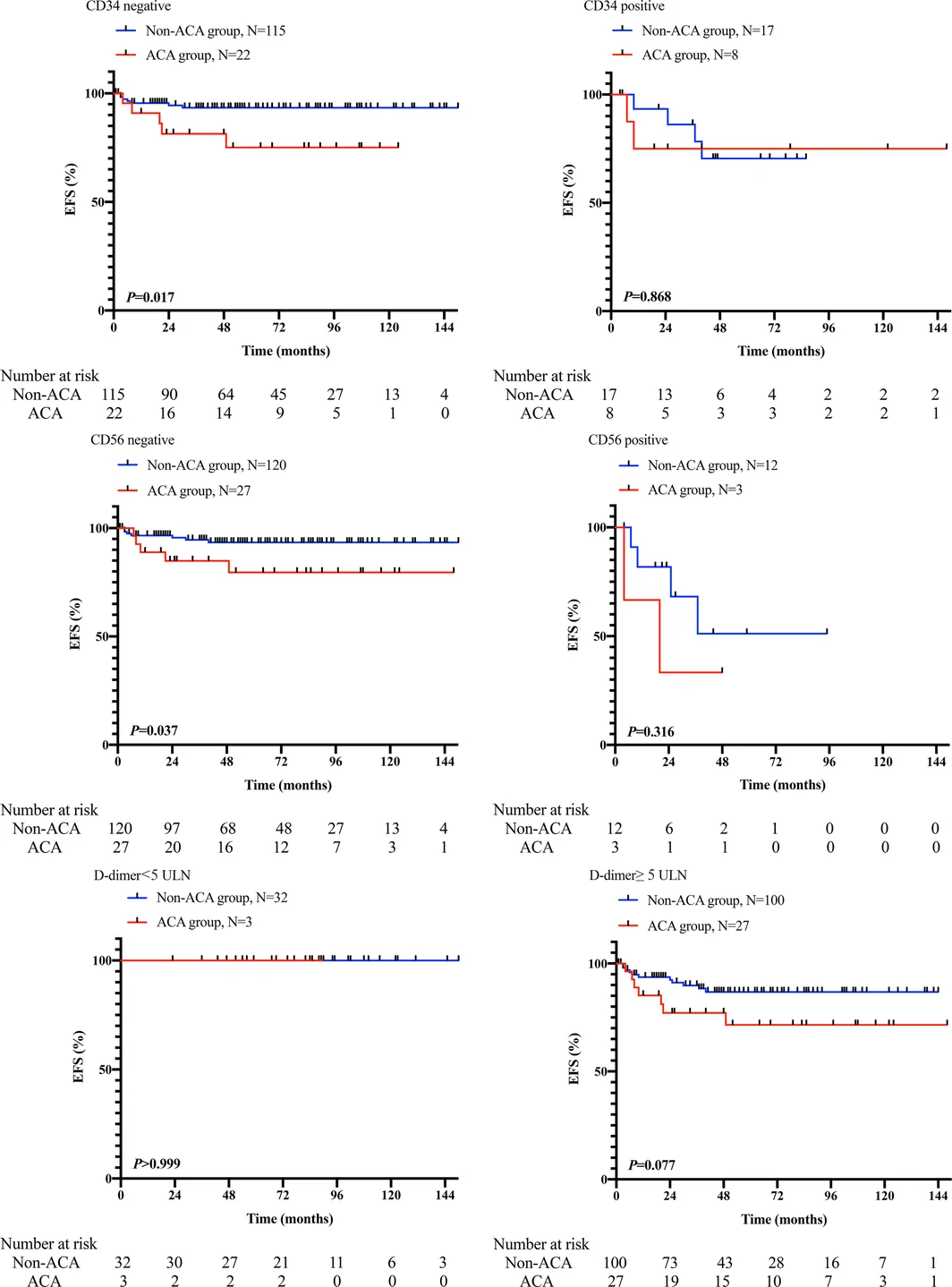
Subgroup analysis was performed to further explore the prognostic significance of ACA in APL patients. EFS, event‐free survival; OS, overall survival; PFS, progression‐free survival.

To further explore the prognostic significance of ACA in EFS, subgroup analysis was performed for prognostic factors (D‐dimer, CD34, and CD56 expression) other than ACA in multivariate analysis. The study showed that in CD34 and CD56‐negative expression groups, the EFS of the ACA group was significantly worse than that of the non‐ACA group (*p* = 0.017; *p* = 0.037) (Figure 3).

## 4 DISCUSSION

Our study showed that roughly one fifth of patients (19.3%) have ACA besides the *t* (15;17), somewhat lower than the ranges reported in the literature. The presence of ACA was associated with lower hemoglobin levels and a higher risk stratification. This observation suggests that during induction remission, better supportive care should be given to patients with ACA, such as keeping hemoglobin higher level actively, and decreasing WBC counts effectively. Multivariate analysis indicated that ACA remained a potentially unfavorable prognostic factor for EFS. In CD34 and CD56 negative APL, patients harboring ACA had inferior EFS by further subgroup analysis.

One noteworthy difference between our results and published reports was the study definition of EFS. In this observation, we defined EFS as time from mCR to any event, including SPM, QT prolongation, internal bleeding, disease recurrence, and death. Compared with widely used criteria (from diagnosis to death or relapse), calculated time and involved events are two distinct differences. Now, benefiting from dramatic advances in the outcome, APL has become the most curable subtype of acute myeloid leukemia.^16^ Despite challenges still remaining, after molecular remission, long‐term survival and high quality of life have become the ultimate goal. Not only death or relapse but any event should also be given full attention. In this respect, ATO‐related side effects, SPM, and disease recurrence have been a major focus of attention. The adverse effects of ATO are usually reversible by temporary discontinuation and early intervention. Nevertheless, some complications such as gastrointestinal bleeding and cardiotoxicity can induce life‐threatening even death outcomes.^17^ Although successful therapy, some APL survivors still face the potential problem of developing SPM and disease relapse, and a minority of patients ultimately died of these events.^18^, ^19^, ^20^ So far, disappointingly, foreign co‐workers and our studies had not found any predictive factor for SPM, moreover, the SPM risk can increase over time.^21^ Given that, we redefined the criteria of EFS. Measured time was from mCR achievement, involved events included QT prolongation, internal bleeding, SPM, disease recurrence, and death.

Our study showed that ACA was an independent adverse factor for EFS. Patients harboring ACA more frequently complicated QT prolongation, bleeding, SPM, disease recurrence, and death. For these “high‐risk” subjects, preventive strategies such as careful monitoring of electrocardiogram and electrolytes, regular screening of cancer markers, imaging and endoscope, and close detection of the *PML::RARα* gene should be emphasized to allow early diagnosis and intervention. While initial ACA can exactly provide “warning” information for inferior EFS, this is especially necessary for CD34 and CD56 negative expression patients with favorable prognosis.

There are several limitations to our study. First, a relatively small sample size and a relatively low incidence might have underpowered our analysis. Second, although all patients received the ATRA‐ATO combination regimen, the additional components of therapy might have an impact on clinical outcomes. Further prospective or retrospective studies should include sufficient numbers of uniformly treated patients to garner greater statistical power.

In summary, our results highlighted the importance of pursuing full karyotype analysis rather than relying solely on PCR‐ or FISH‐based detection. To our knowledge, this study is the first to have involved the most study parameters. Concerning disease variables, besides lower hemoglobin levels and higher risk stratification, we did not demonstrate significant differences between ACA and non‐ACA groups. With regard to clinical outcomes, we first reported the relationship between ACA with some events such as SPM, QT prolongation, and embolism. As for the impact on survival endpoints, our observation did not indicate that ACA had an influence on OS and PFS, but our study confirmed ACA carried prognostic importance and conferred inferior EFS.

## AUTHOR CONTRIBUTIONS

**Hui Zeng:** Conceptualization (equal); data curation (equal); formal analysis (equal); investigation (equal); methodology (equal); software (equal); writing – original draft (equal). **Hai‐Bo Dong:** Conceptualization (equal); data curation (equal); formal analysis (equal); investigation (equal); methodology (equal); software (equal); writing – original draft (equal). **Qi‐Guo Zhang:** Investigation (supporting); methodology (supporting); resources (supporting); supervision (supporting); writing – review and editing (supporting). **Min Zhou:** Data curation (supporting); resources (supporting); software (supporting); supervision (supporting). **Qian Zhang:** Data curation (supporting); resources (supporting); software (supporting). **Lan‐Xin Chen:** Data curation (supporting); resources (supporting); software (supporting). **Cui‐Ying Yuan:** Data curation (supporting); resources (supporting); software (supporting). **Ru‐Ru Jiang:** Data curation (supporting); resources (supporting); software (supporting). **Jin‐Wen Liu:** Data curation (supporting); resources (supporting); software (supporting). **Jian Ou‐Yang:** Data curation (supporting); formal analysis (supporting); resources (supporting); software (supporting); supervision (supporting). **Jie He:** Formal analysis (supporting); investigation (supporting); methodology (supporting); supervision (equal); writing – review and editing (equal). **Bing Chen:** Resources (supporting); software (supporting); supervision (equal); validation (supporting); visualization (supporting); writing – review and editing (equal).

## CONFLICT OF INTEREST STATEMENT

All authors have declared that no competing interest exists.

## ETHICS APPROVAL STATEMENT

This retrospective study was approved by the Ethics Committee of Nanjing Drum Tower Hospital (2023–220‐01).

## Data Availability

All data is available in the medical records departments of Nanjing Drum Tower Hospital.
